# Supplementary material for: Patterns of Intron Gain and Loss in Fungi
Source: PLoS Biol. 2004 Nov 30;2(12):e422. doi: 10.1371/journal.pbio.0020422 (PMC532390; doi:10.1371/journal.pbio.0020422)
Supplement: Table S1 — Also available at http://genes.mit.edu/NielsenEtAl/. (4.3 MB ZIP). [file pbio.0020422.st001.zip › NielsenEtAl/html/545.html]

AN6225.1.NCU05313.1.MG06075.1.FG05314.1


```
 CLUSTAL W (1.82) Multiple Sequence Alignments - Introns Inserted


Sequence 1: NCU05313.1	153 aa
Sequence 2: MG06075.1	154 aa
Sequence 3: FG05314.1	153 aa
Sequence 4: AN6225.1	153 aa
Alignment Length: 155 aa
Number Identitical Residues: 97 aa
Alignment Score (without introns) 4371


MG06075.1 	-MGTNLPY1AIDAET2PLQPAELAVLRAQYDKEGDMVGVQTKFNYAW0GLVKSSTRSDQQ
NCU05313.1	--MTQLPY1AVDAET2PLNPAELNVLRAQYEKEGEMVGVQTKFNYAW0GLVKSNVRADQH
FG05314.1 	-MVTELPY1ALDAET2PLNPSELNVLKAQYDREGEMVGVQTKFNYAW0GLVKSNQRNDQQ
AN6225.1  	MTSSNLPY1AADAES2PLKPAELQVLRAQYEKEGDYVGVQTKFNYAW0GLIKSNFRADQQ
          	   ::*** * ***: **:*:** **:***::**: *********** **:**. * **:

MG06075.1 	LGVMLLSEIFRTSPERRRECLYYLALGNYKLGNYAEARRYNDLLLGKEPTNLQATNLRSL
NCU05313.1	LGVMLLSEIFRTSPERRRECLYYLALGNYKLGNYAQARKYNDALLENEPANLQAANLRAL
FG05314.1 	LGVRLLSDIFRVSPERRRECLYYLALGNYKLGNYGEARRYNDLLLDKEPANLQASNLRSL
AN6225.1  	EGVRLLSEIFRAHPERRRECLFYLALGNYKLGNYGEARRYNDLLIDKEPGNLQAASLRTL
          	 ** ***:***. ********:************.:**:*** *: :** ****:.**:*

MG06075.1 	IDDKVAKEGLLGAAIIGGVAVAASVVGGILIRNMGRKR
NCU05313.1	IDDKVTKEGLMGVAIISGVAVAAGVIGGVLLRNLGRKR
FG05314.1 	IDDKVAREGLMGVAILSGVGVAAGVVGAFLLRN-ARKR
AN6225.1  	IDDRVSREGMVGIAIVGGLALAAGVVGGLLFRG--QRR
          	***:*::**::* **:.*:.:**.*:*..*:*.  ::*
```
